# H3K4 Methyltransferase CfSet1 Is Required for Development and Pathogenesis in *Colletotrichum fructicola*

**DOI:** 10.3390/jof8040363

**Published:** 2022-04-01

**Authors:** Yalan Gao, Shengpei Zhang, He Li

**Affiliations:** 1 Key Laboratory of National Forestry, Grassland Administration on Control of Artificial Forest Diseases and Pests in South China, Central South University of Forestry and Technology, Changsha 410004, China; 20201100019@csuft.edu.cn (Y.G.); T20182400@csuft.edu.cn (S.Z.); 2 Hunan Provincial Key Laboratory for Control of Forest Diseases and Pests, Central South University of Forestry and Technology, Changsha 410004, China

**Keywords:** *Camellia oleifera*, *Colletotrichum fructicola*, methyltransferase, CfSet1, pathogenicity

## Abstract

Tea-oil tree (*Camellia oleifera* Abel.) is a unique woody edible oil species in China. Anthracnose is the common disease of *Ca.* *oleifera*, which affected the production and brought huge economic losses. *Colletotrichum fructicola* is the dominant pathogen causing *Ca.* *oleifera* anthracnose. The gene *CfSET1* was deleted and its roles in development and pathogenicity of *C. fructicola* were studied. Our results show that this protein participated in the growth, conidiation, appressorium formation, and pathogenicity of this fungal pathogen. Our results help us understand the mechanisms of pathogenesis in *C. fructicola* and suggest CfSet1 as a potential target for the development of new fungicide.

## 1. Introduction

Tea-oil tree (*Camellia oleifera*) is native to China, and has been cultivated for more than 2300 years, mainly for its high-quality cooking oil. However, the fungal disease anthracnose has caused a serious yield reduction in the seeds of tea-oil trees. Anthracnose typically results in a 10~30% reduction in tea oil each year, and the severely affected areas often experience more than a 50% of loss of tea oil [1]. Previous studies have demonstrated that *Colletotrichum fructicola* is the dominant pathogen causing *Ca. oleifera* anthracnose [2]. 

Histone methylation and acetylation modification are two common mechanisms of post-translational modification. Zhang et al. [3] have previously revealed that a histone acetyltransferase CfGcn5 regulates growth, development, and pathogenicity in the anthracnose fungus *C. fructicola* on tea-oil trees. However, the function of histone methylation in *C. fructicola* remains unknown. In fungi, methylation is involved in a variety of biological processes, such as development, substrate utilization, and pathogenicity. In *Saccharomyces cerevisiae*, histone 3 lysine 4 methylation (H3K4me) requires H3K4 methyltransferase (complex of proteins associated with Set1 (COMPASS)) which is composed of *SET1*/*KMT2* and other proteins [4]. Dallery et al. [5,6] previously demonstrated that H3K4 trimethylation by CclA encoding one COMPASS subunit regulates development, pathogenicity, and secondary metabolism in *C. higginsianum*.

Here, we investigated the biological function of H3K4 methyltransferase CfSet1 in *C. fructicola,* which orchestrates growth, development, and pathogenicity. 

## 2. Materials and Methods

### 2.1. Test Strain

The wild-type (WT) strain, CFLH16, was isolated from a tea-oil tree field and identified in the China Ministry of Education key laboratory for non-wood forest cultivation and conservation. It represents the dominant genotype of the main species *C. fructicola* responsible for anthracnose in tea-oil trees. The *CfSET1* deletion mutants and complemented strains were obtained as described below in this research.

### 2.2. CfSet1 Sequence Analysis

The Set1 protein (NP.011987.1) of *S. cerevisiae* was used as a query to search for its homolog in the *C. fructicola* genome database by BLASTP. In addition, Set1 proteins in *Colletotrichum camelliae*, *Colletotrichum aenigma*, *Beauveria bassiana*, *Neonectria ditissima*, *Fusarium poae, Colletotrichum siamense*, *Verticillium nonalfalfae*, and *S. cerevisiae* were acquired from the NCBI database (https://www.ncbi.nlm.nih.gov/, accessed on 22 January 2022). The phylogenetic tree showing the relationships among Set1 proteins from these species was constructed by MEGA 7.0 programs using a neighbor-joining method with 1000 bootstrap replicates.

### 2.3. Generation of CfSET1 Deletion and Complemented Strains

#### 2.3.1. Obtaining of *CfSET1* Deletion Strains

Based on the principle of homologous recombination, the genomic DNA of wild-type strain CFLH16 was used as a template to amplify the upstream and downstream coding region of *CfSET1* by polymerase chain reaction (PCR) with primer pairs for Set1-1F/Set1-2R and Set1-3F/Set1-4R (about 1 kbp), respectively. The hygromycin phosphotransferase gene (HPH) fragment (about 1.4 kb) was amplified using Hyg-F/Hyg-R primers. Then, the upper and lower arms and HPH fragments were used as templates, and the *CfSET1* gene deletion fragment was amplified by over-lap PCR using Set1-1F/Set1-4R primers. The recombinant deletion fragment was transformed into the protoplasts of the wild-type strain of *C. fructicola* by polyethylene glycol (PEG) method [7]. The hygromycin-resistant transformants were screened by PCR amplification with in-gene primer pair Set1-7F/Set1-8R and out-of-arm primer pair Set1-5F/H885R and verified by electrophoresis. The transformants that could not amplify the target band with primers Set1-7F/ Set1-8R but amplified the target band with primers Set1-5F/H885R were *CfSET1* deletion mutants. Through screening, we will identify Δ*Cfset1* mutants.

#### 2.3.2. Obtaining Complemented Strains of CfSET1 Deletion Mutant

The promoter and coding genes of *CfSET1* were amplified by PCR using the primers Set1-9F/Set1-10R and Set1-11F/Set1-12R. The GFP gene fragment was amplified using primers GFPF/GFPR. The target gene fragment was amplified by PCR fusion using primers Set1-9F/Set1-12R. The promoter-GFP-target gene fragment was amplified with PCR connection using Set1-9F/Set1-12R primers. The PCR product was purified and co-transferred with linear pYF11 plasmid into yeast XK-125 competent cells to form the pYF11::*CfSET1*-complemented vector. The yeast cells were spread on SD-Trp solid plates for screening and incubated for 3 days at 28 °C under dark conditions. Using transformed yeast as a template, primers Set1-9F/GFPR were used for PCR identification of positive clones. Validated yeasts were cultured in liquid YPD medium in a shaker for 12 h. The successfully transformed plasmids were extracted and transferred into competent cells of *Escherichia coli* JM109. The transformed *E. coli* cells were spread on LB plates and cultured at 37 °C under dark conditions for 3 days and then transferred into liquid LB medium. The *E. coli* plasmids were extracted from the liquid culture and verified using primer Set1-9F/GFPR and sequenced. The plasmids with correct sequence for genetic complementation were transferred into Δ*Cfset1*-20 protoplasts of *C. fructicola* by the PEG method. The transformants that could grow on media containing bleomycin were verified by green fluorescence examination. Through screening, we will identify the complemented strains. All primers used in this work are listed in Table 1.

### 2.4. Phenotypic Assays of the CfSet1 Deletion Mutants

The biological phenotypes of CFLH16, the Δ*Cfset1*-20 mutant, the Δ*Cfset1*-28 mutant, and the complemented Δ*Cfset1*/*SET1* strains were measured for growth and development, appressorium formation, and pathogenicity. Three replicates of each strain were used for each phenotype assay and the experiment was repeated three times.

#### 2.4.1. Growth Rate Determination

Agar blocks (Φ = 8 mm) containing mycelia of strains CFLH16, the Δ*Cfset1*-20 mutant, the Δ*Cfset1*-28 mutant, and Δ*Cfset1*/*SET1* were cultured in the plates of potato dextrose agar (PDA) (dextrose: Sinopharm Chemical Reagent Co., Ltd. in Shanghai, China; agar: Life Sciences) for 3 days, and colony diameters were measured. 

#### 2.4.2. Asexual Conidia Assays

Mycelia of strains CFLH16, the Δ*Cfset1*-20 mutant, the Δ*Cfset1*-28 mutant, and Δ*Cfset1*/*SET1* were cultured in liquid shaking PDB for 3 days. Then, conidiation was quantitatively determined and conidia morphology was observed under microscope. 

#### 2.4.3. Appressorium Formation and Penetration Assays

The conidia of strains CFLH16, the Δ*Cfset1*-20 mutant, the Δ*Cfset1*-28 mutant, and Δ*Cfset1*/*SET1* were washed three times with sterile water, and the concentration of spore suspension was adjusted to approximately 1 × 10^5^ spores/mL. Then, 10 μL of spore suspension was incubated on the center of hydrophobic slides at 28 °C for 16 h. Similarly, 10 μL of spore suspension was incubated on the inner epidermis of white onion at 28 °C for 5 days. Then, the rates of appressorium formation and appressorial invasion were statistically analyzed.

#### 2.4.4. Pathogenicity Assays

Mycelial blocks of strains CFLH16, Δ*Cfset1*-20, Δ*Cfset1*-28, and Δ*Cfset1*/*SET1* (Φ = 8 mm) were inoculated onto the abaxial edge of unwounded tea-oil tree leaves. The inoculated leaves were incubated in darkness at 28 °C with 100% humidity for 4~5 days and then photographed. The sizes of the lesions were measured.

## 3. Results and Discussion

### 3.1. Identification and Phylogenetic Tree Analysis of CfSet1

Based on the ScSet1 protein (NP.011987.1) of *S. cerevisiae*, a homologous protein was identified in the *C. fructicola* genome by BLASTP analysis and named CfSet1 (XP_007274068.1). The protein consists of 1270 amino acids. The core SET domain of the protein was surrounded by pre-SET (N-SET) and post-SET domains on both sides. The protein also contains six unknown structural domains (Figure S1a in Supporting Information). 

Phylogenetic analysis revealed that CfSet1 shared a high amino acid sequence identity with that of *Ca. camelliae* (identify: 97.8%) and a low but significant sequence identity with *S. cerevisiae* Set1 (identify: 50.9%) (Figure S1b in Supporting Information). This result indicates that Set1 proteins are well conserved among the analyzed fungi. 

To address the function of the CfSet1 protein, we obtained two *CfSTE1* gene deletion mutants Δ*Cfset1-20* and Δ*Cfset1-28*, which were confirmed by diagnostic PCR analysis. In addition, we also obtained the complemented strain Δ*Cfset1*/*SET1* with a wild-type *CfSTE1* gene. These and the green fluorescent protein (GFP)-encoding gene were fused together and transformed into the ΔCfset1-20 background strain (Figure S2 in Supporting Information).

### 3.2. CfSet1 Is Involved in Vegetative Growth and Asexual Reproduction of C. fructicola

**Figure 1:**
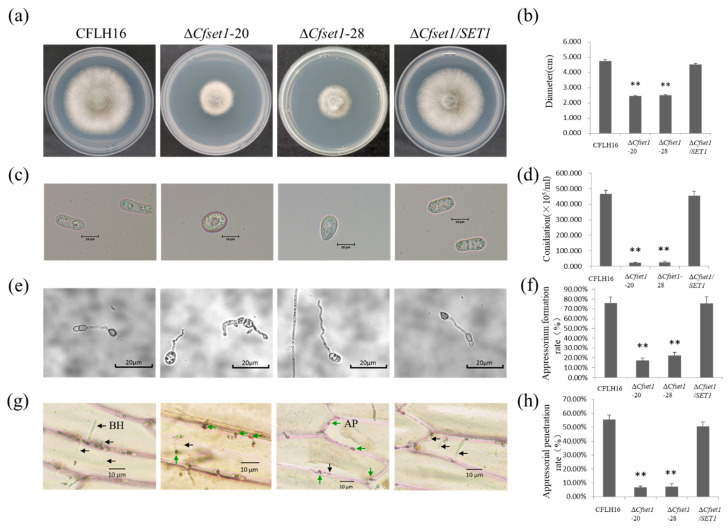
Vegetative and reproductive growth as well as appressorium formation of *C. fructicola*. (**a**) Growth rate of mutants Δ*Cfset1*-20 and Δ*Cfset1*-28 were significantly reduced. (**b**) Statistical analysis of the colony diameter. (**c**) Conidial morphology of Δ*Cfset1* mutants were larger and more spherical than CFLH16 and Δ*Cfset1*/*SET1*. (**d**) Conidiation of mutant strains Δ*Cfset1*-20 and Δ*Cfset1*-28 were significantly reduced. (**e**) Appressorium formation of mutant strains Δ*Cfset1*-20 and Δ*Cfset1*-28 were significantly reduced. (**f**) Statistical analysis of the rate of appressorium formation on hydrophobic slides. (**g**) The biotrophic hyphae of mutant strains Δ*Cfset1*-20 and Δ*Cfset1*-28 were significantly reduced. Green arrows: appressorium (AP). Black arrows: biotrophic hyphae (BH). (**h**) Statistical analysis of appressorial penetration rate. Double asterisks indicate that each mutant has significant differences with wild-type and complemented strains (*p* < 0.01).

To examine the role of CfSet1 in vegetative growth, the growth rate of Δ*Cfset1*-20 and Δ*Cfset1*-28 on PDA were investigated and compared with those of CFLH16 and Δ*Cfset1*/*SET1*. Both Δ*Cfset1*-20 and Δ*Cfset1*-28 grew significantly slower than CFLH16 and Δ*Cfset1*/*SET1* (average diameter: CFLH16: 4.77 cm, Δ*Cfset1*-20: 2.45 cm, Δ*Cfset1*-28: 2.52 cm, Δ*Cfset1*/*SET1*: 4.55 cm) (Figure 1a,b). The results reveal that *SET1* is a significant contributor to vegetative growth in *C. fructicola*. 

Asexual conidia play an important role in fungal infection [8]. In the present investigation, we analyzed the conidiation of mutant strains Δ*Cfset1*-20 and Δ*Cfset1*-28. We found that the conidia in Δ*Cfset1* mutants were larger and more spherical than CFLH16 and Δ*Cfset1*/*SET1* (Figure 1c). Furthermore, fewer conidia were produced than the wild-type and complemented strains (Figure 1d). Together, the results show that a loss of *SET1* affected conidia morphology and significantly reduced spore-producing ability.

These findings indicate that CfSet1 is involved in vegetative growth and asexual reproduction in *C. fructicola*. 

### 3.3. CfSet1 Is Required for Appressorium Formation and Penetration of C. fructicola

To understand the role of CfSet1 in appressorium formation and penetration, the appressorium of strains Δ*Cfset1*-20 and Δ*Cfset1*-28 was investigated. We found that the rate of appressorium formation of Δ*Cfset1*-20 was only 17% and Δ*Cfset1*-28 was 22.5% (Figure 1e,f). We further investigated the appressorium-mediated penetration on onion epidermis and found that most Δ*Cfset1* appressoria failed to penetrate epidermal cells, and that only the few penetrated appressoria produced BH. Our statistics show that the appressorial penetration rate of Δ*Cfset1*-20 was only 6.7% and Δ*Cfset1*-28 was 7.3% (Figure 1g,h). Both ratios were significantly reduced from those formed by strains CFLH16 and the Δ*Cfset1*/*SET1*. These results indicate that CfSet1 is required for appressorium formation and penetration of *C. fructicola*. 

### 3.4. CfSet1 Is Important for Pathogenicity of C. fructicola

**Figure 2:**
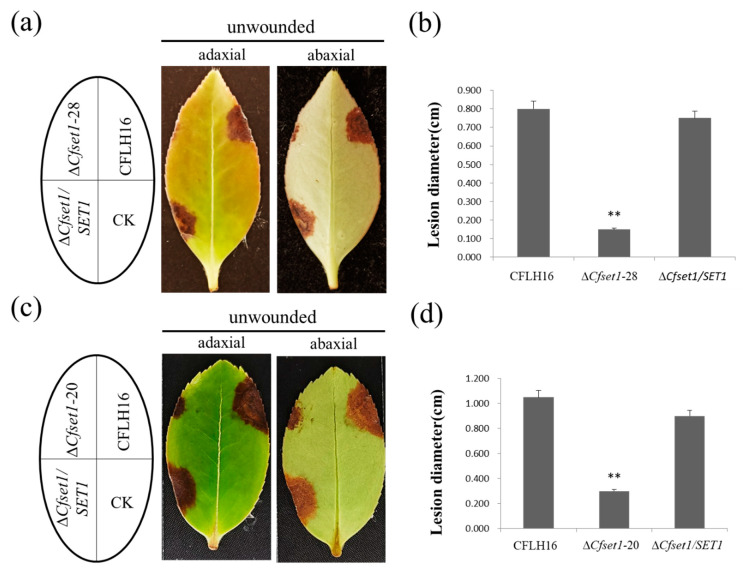
Pathogenicity of *CfSET1* gene-deleted mutants to tea-oil leaves. (**a**,**c**) The pathogenicity of Δ*Cfset1-*20 and Δ*Cfset1-*28 was significantly weaker than both CFLH16 and Δ*Cfset1*/*SET1*. The tested strains caused yellow-brown, *irregular*-shaped lesions on tea-oil leaves. CK: blank control. (**b**,**d**) Statistical analysis among three strains for their lesion sizes on unwounded leaves. Double asterisks indicate each mutant has significant differences with wild-type and complemented strains (*p* < 0.01).

The appressorium formed by conidium is a pivotal structure for host infection by plant fungal pathogens. Our results show that the *CfSET1* gene participated in appressorium formation in *C. fructicola*, so we investigated the pathogenicity of more representative mutant strains. After four days, the mutant strain Δ*Cfset1*-28 caused a lesion area with an average diameter of 0.15 cm on fresh tea-oil leaves (Figure 2a,b). The mutant strain Δ*Cfset1*-20 caused a lesion area with an average diameter of 0.3 cm on fresh tea-oil leaves (five days, Figure 2c,d). Both lesion sizes were significantly smaller than those caused by strains CFLH16 and Δ*Cfset1*/*SET1*. These results indicate that CfSet1 is important to the pathogenicity of *C. fructicola*.

In this study, CfSet1, an H3K4 methyltransferase, was identified in *C. fructicola* and its biological function was investigated. The COMPASS is conserved from yeast to multicellular eukaryotes, of which Set1 plays a vital role in plant pathogen. In *S. cerevisiae*, *B. bassiana*, and *Magnaporthe oryzae*, *SET1* gene deletion mutants showed a significant reduction in mycelial growth rate [9,10,11]. In addition, Set1 plays an important role in conidiation and conidial quality control in *B. bassiana* and *M. oryzae* [9,10,11]. Our findings show that the loss of *CfSET1* significantly reduced the growth rate and the spore-producing ability of the Δ*Cfset1* mutants, indicating that CfSte1 has a conserved function in regulating growth and conidiation among fungi. Appressoria play a vital role in pathogen invasion during host–pathogen interaction. For example, to infect rice plants, the blast fungus *M. oryzae* produces appressoria, which rupture the leaf cuticle and allow fungal hyphae to invade and colonize the host tissues [12]. The strongly reduced appressorium formation rate of the Δ*Cfset1* mutants indicates that CfSet1 participated in appressorium formation, which might forecast its roles in pathogenicity. Our results also indicate that CfSet1 is indeed important for pathogenicity of *C. fructicola*, which is also supported by the results in *B. bassiana* and *M. oryzae* [10,11]. Taken together, the Δ*Cfset1* mutants showed low pathogenicity on tea-oil leaves, which is mainly caused by decreased conidiation, appressorium formation, and penetration. Furthermore, there are other potential mechanisms affecting pathogenicity that need further investigation.

In summary, a *S. cerevisiae ScSET1* homologous gene *CfSET1* was identified and disrupted from *C. fructicola*. Our analyses show that *CfSET1* plays a key role in growth, conidiation, appressorium formation, and pathogenicity, which should encourage further exploration into the characterization of *CfSET1*. These results are useful for elucidating the pathogenic molecular mechanisms of *C. fructicola* and for identifying a potential target for the development of new fungicide.

## Figures and Tables

**Table 1:** Primers used in this study for amplifying related gene fragments.

Primer	Primer Sequence (5′→3′)	Purpose
Set1-1F	GCAGCCAAGGCTTGTATGAA	amplify *CfSET1* 5′ flank sequence
Set1-2R	TTGACCTCCACTAGCTCCAGCCAAGCCCGTGAGGTGTATCTGTCTCT	amplify *CfSET1* 5′ flank sequence
Set1-3F	CAAAGGAATAGAGTAGATGCCGACCGTCCTGATCGCTTCTTTCCGG	amplify *CfSET1* 3′ flank sequence
Set1-4R	GCTATCAGATAAAGTCCCGT	amplify *CfSET1* 3′ flank sequence
Set1-5F	TGAGCCTACAATATCACGAC	validation of *CfSET1* gene deletion
H855R	GCTGATCTGACCAGTTGC	validation of *CfSET1* gene deletion
Set1-7F	TGCCTACCGACTTCAAGCTG	amplify *CfSET1* gene sequence
Set1-8R	GGTCCAAACTGCCGATCTCA	amplify *CfSET1* gene sequence
Set1-9F	ACTCACTATAGGGCGAATTGGGTACTCAAATTGGTTACTAGGCCTGCCAGAGCAGC	amplify complemented sequence
Set1-10R	CCTCGCCCTTGCTCACCATCGTGAGGTGTATCTGTCTCT	amplify complemented sequence
Set1-11F	GCATGGACGAGCTGTACAAGATGACCCGCCAACCGTCGGC	amplify complemented sequence
Set1-12R	CACCACCCCGGTGAACAGCTCCTCGCCCTTGCTCACTTAGTTGAGGAATCCCTTGC	amplify complemented sequence
GFPF	ATGGTGAGCAAGGGCGAGG	amplify *GFP* gene sequence
GFPR	CTTGTACAGCTCGTCCATGC	amplify *GFP* gene sequence
Hyg-F	CTCTATTCCTTTGCCCTCG	amplify *HPH* gene sequence
Hyg-R	GCTGATCTGACCAGTTGC	amplify *HPH* gene sequence

## Data Availability

All data generated or analyzed during this study are included in this published article and its supplementary additional files.

## Supplementary Materials

The following supporting information can be downloaded at: https://www.mdpi.com/article/10.3390/jof8040363/s1. Figure S1: Phylogenetic analysis and domain prediction of CfSet1. (a) The purple oval indicates the pre-SET (N-SET) domain, the brown pentagon represents the core SET domain, the gray quadrilaterals represent post-SET, and the pink and green boxes refer to six low-complexity regions. (b) The neighbor-joining tree was constructed by MEGA 7.0 with 1000 bootstrap replicates. Figure S2: The green fluorescence of complemented strain Δ*Cfset1/SET1*.
