# Supplementary material for: H3K4 Methyltransferase CfSet1 Is Required for Development and Pathogenesis in Colletotrichum fructicola
Source: J Fungi (Basel). 2022 Apr 1;8(4):363. doi: 10.3390/jof8040363 (PMC9025643; doi:10.3390/jof8040363)
Supplement: Supplementary file 1 [file jof-08-00363-s001.zip › jof-1624169-supplementary.pdf]

Supporting information

The H3K4 Methyltransferase CfSet1 is Required for Development and Pathogenesis in *Colletotrichum fructicola* Gao Yalan et al.

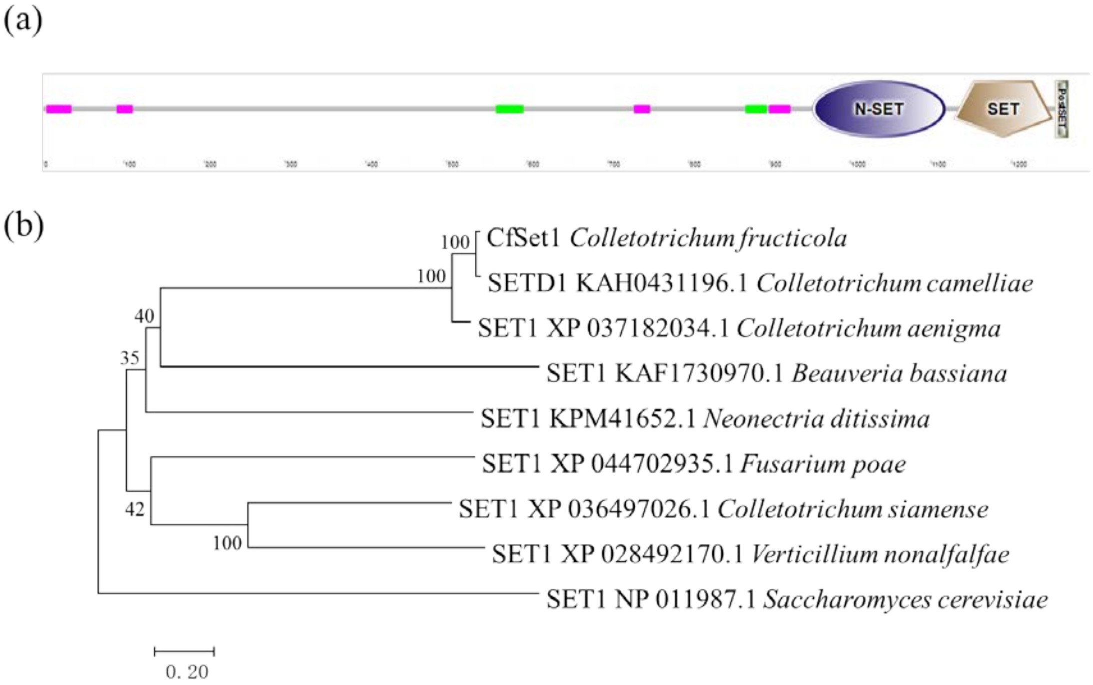

Figure S1 Phylogenetic analysis and domain prediction of CfSet1. a)The purple Oval indicates the Pre-SET (N-SET) domain, the brown pentagon represent the core SET domain, the gray quadrilaterals represent Post-SET as well as pink and green boxes refer to six low complexity regions. b) The neighbour-joining tree was constructed by MEGA 7.0 with 1000 bootstrap replicates.

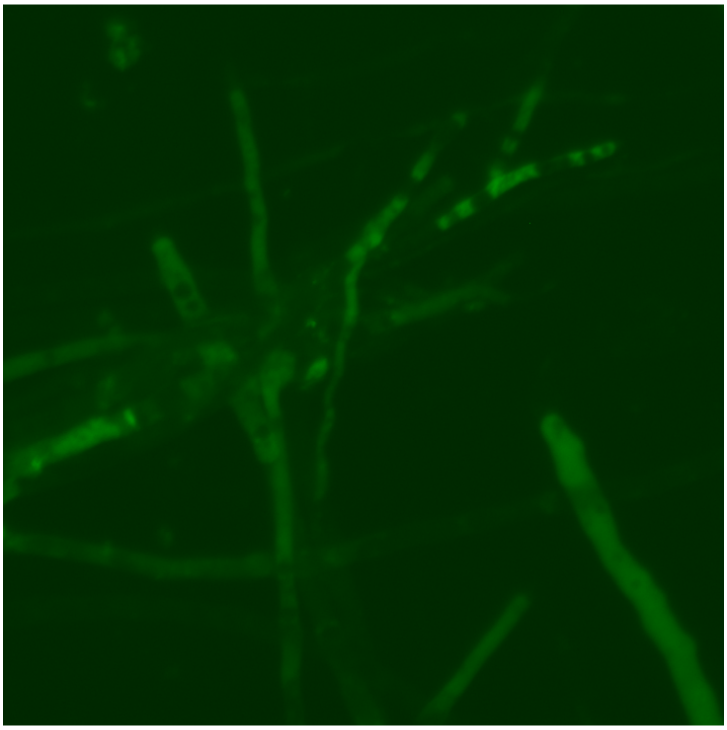

Figure S2 The green fluorescence of complemented strain Δ*Cfset1/SET1*.
